# Evolution over the last 40 years of the assisted reproduction technologies in cattle - the Brazilian perspective I - timed artificial insemination

**DOI:** 10.1590/1984-3143-AR2024-0034

**Published:** 2024-08-12

**Authors:** José Nélio de Sousa Sales, Guilherme Pugliesi, Laís Reis Carvalho, Luiz Manoel Sousa Simões, Lucas Araujo Lemos, Matheus Pedroso Vicente, Rafael Resende Rabelo Silva, Pietro Sampaio Baruselli

**Affiliations:** 1 Departamento de Medicina Veterinária, Universidade Federal de Juiz de Fora, Juiz de Fora, MG, Brasil; 2 Faculdade de Zootecnia e Medicina Veterinária, Universidade Federal de Lavras, Lavras, MG, Brasil; 3 Departamento de Reprodução Animal, Faculdade de Medicina Veterinária e Zootecnia, Universidade de São Paulo, Pirassununga, SP, Brasil; 4 Faculdade de Medicina Veterinária e Zootecnia, Universidade de São Paulo, São Paulo, SP, Brasil

**Keywords:** ovulation, estrus, synchronization, P4

## Abstract

The reproductive efficiency of the herd is correlated with higher productivity in livestock. Reproduction biotechniques, such as ovulation synchronization protocols, are important to optimize production and accelerate genetic profit in beef and dairy herds. The objective of this review is to describe the evolution over the last 40 years of the artificial insemination (AI) and the timed-AI (TAI) protocols in cattle from a Brazilian perspective. TAI protocols are based on synchronizing emergence of the wave of follicular growth, controlling circulating progesterone (P4) concentrations, stimulating the final growth of the follicle and inducing a synchronized ovulation. Hormonal alternatives that optimize the response at the end of the protocol and strategies to induce final follicle growth and ovulation in categories of females with low expression of estrus are described. Furthermore, the potential positive effect of previous exposure to injectable P4 on fertility of *Bos indicus* and *Bos taurus* cows is also discussed.

## Introduction

In the last 40 years, assisted reproductive technologies (ARTs) have transformed the dairy and beef production industries leading to significant improvements in the quality and quantity of animal products. Among them, artificial insemination (AI) and embryo transfer (ET) are the most widely used biotechnologies (Baruselli et al., 2019). In recent years, a significant increase in the use of AI has been observed due to the development of timed-AI (TAI) protocols which allowed AI of cyclic and anestrus cows and heifers to be performed at a pre-determined time, without the necessity of estrus detection (Baruselli et al., 2017).

The first TAI protocol developed was based on the association of prostaglandin F2α (PGF2α) and GnRH (Pursley et al., 1995). Denominated *Ovsynch*, this protocol consisted of the administration of GnRH on a random day of the estrous cycle, with the aim of inducing ovulation and the consequent emergence of a new follicular wave. Furthermore, 7 days later, a dose of PGF2α was administered to regress possible corpus luteum (CL). Finally, 48 hours later, a second dose of GnRH was administered to synchronize ovulation. *Ovsynch* is widely used in dairy cows, however, to obtain satisfactory results a high ovulation rate at the first GnRH is necessary. In cows (Vasconcelos et al., 1999) a greater response to the synchronization of ovulation and conception was observed when the protocol was started between days 5 and 12 of the estrous cycle. Thus, pre-synchronization protocols were developed to optimize the response to the first GnRH of *Ovsynch*.

After a few years, some hormonal modifications were carried out in order to improve the final response to the ovulation synchronization protocol. In the early 2000s, a protocol based on P4 and estradiol (E2) was reported (Bo et al., 2003; Baruselli et al., 2004), and is currently the most used in Brazil. The insertion of a P4 intravaginal device simultaneously with administration of E2, at a random moment of the estrous cycle, induces synchronization of the emergence of a new follicular wave (Bo et al., 2003). The period of permanence of the P4 intravaginal device controls follicular growth and ovulation (Prata et al., 2020). After removing the source of P4 (PGF2α and P4 device withdrawal), another hormonal treatment is used to synchronize ovulation (sources of E2 and/or GnRH; Sales et al., 2012). TAI is performed 48 hours after P4 removal. In addition, the use of equine chorionic gonadotropin (eCG) at the time of P4 device removal increased final follicular growth and pregnancy per AI (P/AI) in different categories (Sales et al., 2011a). However, in primiparous cows the results were more pronounced (Sales et al., 2016).

The TAI using E2 and P4 is well-established in suckled *Bos indicus* cows and substantially increases the reproductive efficiency of beef herds, leading to numerous economic benefits related to beef production (Baruselli et al., 2019). The TAI programs reduce the calving interval, increase conception at the beginning of the reproductive period, and increase the number of pregnant cows during the breeding season and the number of calves with greater genetic merit from AI (Baruselli et al., 2018). Due to these characteristics, interest has increased in the use of TAI in beef cows using resynchronization programs (Pugliesi et al., 2019). Thus, the objective of this review was to show the evolution over the last 40 years of the AI and TAI protocols in cattle from a Brazilian perspective.

## Artificial insemination

The majority of bovine herds in tropical and subtropical areas (such as Brazil) are composed of *Bos indicus* because of their greater adaptability to high temperature and humidity, as well as, to the yearly variability in the feed supply (Baruselli et al., 2018). However, a long period of postpartum anestrous is observed in these animals, characterized by follicular emergence sustained by the release of FSH, but compromised final growth of the dominant follicle, and, consequently, absence of ovulation (Baruselli et al., 2018). These changes in the final follicular growth are due to the reduction of LH pulsatility after follicular deviation due to the calf presence and the reduced availability of forage (Jolly et al., 1995; Yavas and Walton, 2000). In cows in which the nutritional requirement is not met because of low feed availability, deficient GnRH secretion and consequently, reduced LH release are observed (Jolly et al., 1995; Montiel and Ahuja, 2005). The reduction of GnRH secretion occurs due to the negative feedback in the hypothalamus promoted by the increase in the concentrations of neuropeptide Y, NEFA, and beta-hydroxybutyrate produced by the mobilization of body fat (Hess et al., 2005). In addition to the nutritional effects, the calf's presence blocks the secretion of GnRH by the hypothalamus through the action of released endogenous opioids (Williams et al., 1996). As a result, AI based on detection of estrus was rarely used in beef and dairy cows managed on pasture.

In 2002, the Brazilian market for AI traded approximately 7.0 million doses of semen, and only 5.8% of dairy and beef females were artificially inseminated. Twenty years later (2023), 24.7 million doses of semen were commercialized and 20 to 23% of the females of the total number of the national herd were inseminated, a huge increase due to TAI (Baruselli, 2024). The increase in the AI market in Brazil occurred simultaneously with the development of the TAI technology. In 2023, 91.2% of females were inseminated by TAI in Brazil. The TAI annual growth rate has been 31.8% in the last 20 years, resulting in great advances and benefits to the meat and milk industries.

### Timed-artificial insemination protocols

Currently, the TAI protocols in beef and dairy cattle are well established, in which P/AI between 30 and 65% are observed (Sales et al., 2015, 2016; Wiltbank et al., 2015; Baruselli et al., 2017). As mentioned before, in *Bos indicus* lactating beef cows, a long period of postpartum anestrus is observed (Baruselli et al., 2004). Due to this physiological condition, the main TAI protocol used in postpartum *Bos indicus* beef cows is based on the association of E2 and P4. Similar protocols are also used in dairy cows in Brazil, contrasting with the protocols based on GnRH and PGF2α that are predominant in the other countries. The standard protocol used based on GnRH and PGF2α is Ovsynch (Pursley et al., 1995). The response (ovulation of the preovulatory follicle at the end) to the TAI protocol based on E2 and P4 is approximately 80% in *Bos indicus* lactating beef cows (Sales et al., 2012) and approximately 85% in dairy cows in GnRH-based protocols and PGF2α when pre-synchronization protocols are used (Souza et al., 2008; Silva et al., 2018). In dairy cows, the Double-Ovsynch protocol has presented a better synchronization result, with ovulation in response to the first GnRH of around 82% and P/AI of 49.7% (Souza et al., 2008). However, some limitations (long protocol of 28 days and too many handlings) may limit the more extensive use of this protocol.

TAI protocols are based on synchronizing the emergence of a new follicular wave either by inducing follicular atresia by the simultaneous administration of P4 and E2 (Baruselli et al., 2017) or ovulating a dominant follicle with a GnRH treatment with at the onset of the TAI protocols (Wiltbank et al., 2015). At the end of a protocol, it is necessary to reduce circulating P4 concentrations by removing the P4 device (exogenous source) and administering PGF2α analog to induce luteolysis (endogenous source), so that, ovulation may occur. Lastly, it is necessary to stimulate the final growth of the preovulatory follicle and to induce a synchronized ovulation (using either GnRH or E2 esters), which allows insemination to be performed at a predetermined moment (Baruselli et al., 2017).

#### Synchronization of follicular wave emergence

Two ways to synchronize the emergence of a follicular wave in the TAI protocols are to promote ovulation of a dominant follicle by administration of GnRH or to induce follicular atresia by the association of P4 and E2.

The Ovsynch protocol (GnRH/PGF2a-based) is by far the most popular among dairy producers (Norman et al., 2009) and uses GnRH to promote ovulation and, consequently, synchronization of wave emergence (Pursley et al., 1995). Although Ovsynch fulfills the three premises for ovulation synchronization, it is not highly efficient (64%) when given at a random day of the estrous cycle (Vasconcelos et al., 1999). Aiming at improving the response to the first GnRH in the Ovsynch protocol, presynchronization protocols have been adopted to increase the proportion of cows within the ideal interval to be started on the protocol, with the largest follicles responsive to the first GnRH (Moreira et al., 2001; Souza et al., 2008). Amongst the presynchronization protocols, Double-Ovsynch has shown the best synchronizing results when compared to Presynch-Ovsynch, with mean ovulation to the first GnRH and P/AI of 82.0% and 49.7%, respectively (Souza et al., 2008). However, there are some limitations with Double-Ovsynch, such as long duration (28 days) and too many cow-handling that may restrict its use. Thus, there is still the need to develop more practical presynchronization protocols. Recently, our research group developed a new presynchronization method by induction of the largest follicle with an intravaginal P4 device 10 days before Ovsynch in lactating crossbred dairy cows, named P4synch (Silva et al., 2018; Sales et al. 2019). In the first study (Silva et al., 2018), P4synch had similar follicular diameter at the time of the 1st GnRH (Double-Ovsynch 17.2±0.7mm and P4synch 18.6±0.9mm; P= 0.28), ovulation rate to the 1st GnRH [Double-Ovsynch 86.3% (44/ 51) and P4synch 81.2% (39/48); P=0.50] and P/AI [Double- Ovsynch 39.0% (89/228) and P4synch 40.1% (85/212); P=0,85]. In the second study (Sales et al., 2019), the pre-synchronization rate (presence the follicle with >12 mm on D0) for P4synch group was 97.8% (45/46). There was difference between groups for presence of CL on D0 (P4E2: 80.4% [37/46] and P4synch: 37.0% [17/46]; P = 0.001), follicular diameter on D0 (P4E2: 15.0 ± 0.8mm and P4synch: 21.0 ± 0.8mm; P = 0.001), at the time ovulation induction (P4E2: 13.9 ± 0.9mm and P4synch: 17.6 ± 0.6mm; P = 0.001) and TAI (P4E2: 15.2 ± 0.7mm and P4synch: 17.2 ± 0.8mm; P = 0.05). Furthermore, there was no difference between groups for synchronization rate (presence de follicle with > 12 mm on TAI; P4E2: 76.1% [35/46] and P4synch: 80.4% [37/46]; P = 0.61), follicular persistence after ovulation induction (P4E2: 8.7% [4/46] and P4synch: 15.2% [7/46]; P = 0.34] and P/AI (P4E2: 37.4% [67/179] and P4synch: 42.4% [72/170]; P = 0.35]. The use of P4-intravaginal devices for periods longer than 10 days results in the development of larger and longer-lasting follicles compared to the natural patterns (Díaz et al., 2015). Cows with P4 devices develop large follicles due to the absence of a pre-ovulatory luteinizing hormone (LH) peak and maintenance of sub-luteal P4 concentrations (Short et al., 1979). Largest follicles are capable of ovulating after long periods (15 days) of P4 blockage (Lucy et al., 1990). Thus, the largest follicle (more than 10 days of growth) may be used in a presynchronization protocol as a more practical tool to precede Ovsynch.

Estradiol esters (E2 benzoate [EB], E2 valerate [EV], and E2 cypionate [EC]) have different pharmacokinetics after administration to animals. Some studies have shown that EB has a shorter half-life and higher peak concentration than EV or CE (Colazo et al., 2003). These pharmacological differences between E2 esters alter reproductive physiology responses during ovulation synchronization protocols (Sales et al., 2012). Some E2 esters are used to induce both synchronization of follicular wave emergence (Bo et al., 2003) and ovulation (Baruselli et al., 2004; Sales et al., 2012). In ovulation synchronization protocols, the most used E2 ester to synchronize follicular wave emergence is EB, and the E2 ester used to synchronize ovulation is EC. The EB can also be used to synchronize ovulation, but additional management is needed during the ovulation synchronization protocol (Sales et al., 2012). Moreover, EV can be used for both synchronization functions and has the advantage of not increasing the number of handlings necessary for the ovulation synchronization protocol (Bo et al., 1993). These differences in the characteristics of E2 esters are related to the size of the ester chain. The longer the ester chain, the lower the solubility in water and the longer the period required for absorption (Mapletoft et al., 2002). Recently, our research group evaluated the effect of EV on follicular dynamics and fertility of lactating *Bos indicus* cows subjected to the ovulation synchronization protocol. In Experiment 1, the occurrence of estrus and P/AI were similar between EB and EV administrated on D0 (P = 0.12 and P = 0.82, respectively). In Experiment 2, P/AI tended to be lower (P = 0.07) in cows inseminated 48 hours after removal of the P4 device when EV was administered at the beginning of the ovulation synchronization protocol compared to TAI at 54 hours. In Experiment 3, the occurrence of estrus (P = 0.12) and P/AI (P = 0.56) were similar between EB and EV administered on D0 and associated with EC on D9 and TAI at 48 hours after P4 device removal (Table 1). Thus, protocols using EV without exogenous ovulation induction require adjustments in the timing of AI from 48 to 54 hours after P4 device removal. However, combining EV at the beginning of the protocol and EC on D9 to induce ovulation allowed TAI to be performed 48 hours after P4 device removal in *Bos indicus* cows. Thus, the use of EV and P4 at the beginning of the ovulation synchronization protocol represents a less expensive alternative (no need to administer PGF2α), with less management (one fewer treatment; less handlings) and with similar reproductive efficiency to of the one with EB in *Bos indicus* cows subjected to TAI protocols. However, EV must be associated with EC so that TAI can be performed 48 hours after the removal of the P4 device (Sales et al., 2024b).

**Table 1:** Effect of estradiol ester used during progesterone-based timed-AI (TAI) protocols in suckled *Bos indicus* cows on the occurrence of estrus and pregnancy per AI (P/AI).

**Variable**	**Occurrence of estrus, % (n/n)**	**P/AI, % (n/n)**	**P**	
			**Estrus**	**P/AI**
Experiment 1				
EB/EC	78.3 (148/189)	52.1 (215/413)	0.17	0.82
EV	72.5 (129/178)	51.8 (207/400)		
Experiment 2				
TAI 48h	73.7 (160/217)	36.4 (79/217)	0.34	0.07
TAI 54h	77.9 (169/217)	45.2 (98/217)		
Experiment 3				
EB/EC	70.9 (156/220)	43.2 (95/220)	0.12	0.56
EV/EC	76.6 (160/209)	45.5 (95/209)		

EB/EC group - 2 mg of estradiol benzoate (EB) on D0 and 265 µg of cloprostenol sodium, 300 IU of equine chorionic gonadotropin (eCG) and 1 mg of estradiol cypionate (EC) on D9. EV group - 5 mg of estradiol valerate (EV) on D0 and 300 IU of eCG on D9. P4 - intravaginal progesterone (P4) device. EV/EC group - 5 mg of EV on D0 and 300 IU of eCG and 1 mg of EC on D9.

#### Circulating P4 concentrations and eCG

During the TAI protocol, it is necessary to control P4 for a certain period to prevent premature ovulation, control the final growth of the dominant follicle, and allow ovulation. To achieve this, the intravaginal P4 device must remain in the females for 7, 8, or 9 days to allow controlled follicular growth and prevent ovulation before AI. After this, it is necessary to drastically reduce circulating P4 to allow synchronized ovulation after administration of the ovulation inducer (Prata et al., 2020). The time of exposure to an intravaginal P4 device depends on the animal category. In primiparous cows, it is necessary to keep the P4 device for a longer period to allow for greater follicular growth, greater ovulation, and improved P/AI. In a recent study (Carvalho et al., 2023), a greater period of permanence (9 days) of P4 device in an E2/P4-based TAI protocol increased [7P4 = 25.0% (47/188) vs 9P4 = 41.1% (79/192); P = 0.01] P/AI in suckled *Bos indicus* primiparous cows. However, in multiparous cows, despite differences in expression of estrus (7D protocol induced lower expression of estrus compared to 8 or 9 D protocols), P/AI was similar among treatment due to the additional effect of GnRH treatment at AI on fertility of females not displaying estrus (Prata et al., 2020).

The use of eCG has shown positive effects on P/AI in herds with anestrous cows, early postpartum cows (less than 2 months postpartum), cattle with inadequate body condition score (<2.75 on a scale from 1 to 5), and in cows with compromised growth of the dominant follicle due to too high circulating P4 concentrations toward the end of the ovulation synchronization treatment (Sales et al., 2011a). It was observed that eCG given on the day of P4 vaginal device removal increased final growth of the preovulatory follicle, neither interfering with the number of ovulatory follicles nor with the time of ovulation. Due to the greater follicle growth, eCG-treated cows had higher ovulation incidence and P/AI. The effects of eCG on follicular dynamics and fertility were observed in multiparous and primiparous cows but were more pronounced in primiparous cows (Sales et al., 2016). In general, eCG increased final growth of the dominant follicle without altering the synchronization of ovulation. Such characteristics allowed for insemination of females at a predetermined time. Similar follicular dynamic results were described previously, in which the final growth of the dominant follicle was significantly greater in eCG-treated cows (1.45 mm/day) compared with control (0.90 mm/day; Sales et al., 2011a). The growth of the dominant follicle is stimulated by eCG due to its affinity for LH and FSH receptors present in follicular granulosa cells (Murphy and Martinuk, 1991). It is known that LH binds to granulosa cell receptors, triggering a cascade of reactions that synthesize catalytic enzymes responsible for producing steroids and consequently stimulate the final growth of the dominant follicle (Carroll et al., 1992). Similarly, eCG would increase E2 production stimulated by the follicular synthesis of cytochrome P450 17A mRNA (Soumano et al., 1998). Thus, eCG would act as a gonadotrophic support, similarly to LH, stimulating the growth of the dominant follicle, especially in low BCS anestrous and primiparous females. In this regard, an interesting strategy to use eCG has been proposed for primiparous *Bos indicus* cows in TAI programs (Pugliesi et al., 2022). In primiparous Nelore cows, splitting the commonly used eCG dose (300IU) over two time points (2 days before and at the time of removal of the P4 device) resulted in a 6.8% increase in P/AI compared to the administration of a single dose at P4 device removal (Pugliesi et al., 2022). Also, in a subsequent study (Sales et al., 2024a), it was observed that splitting the dose or increasing the eCG dose to 400IU positively impacted the P/AI of primiparous cows with BCS ≤ 2.75, but no effects were detected on multiparous cows.

For primiparous cows, an eCG splitting effect was observed on the size of the dominant follicle, as cows receiving eCG in two moments (150 or 200 IU, two times) of the synchronization protocol had a larger follicle and greater P/AI than cows administered eCG only at the time of P4 device removal. In addition, primiparous cows receiving 400 IU eCG, regardless of BCS, had greater P/AI than cows from other treatments. Administering 400 IU to cows with low BCS also resulted in greater P/AI than all other treatments assigned to this category.

#### Synchronization of ovulation

Estradiol esters, such as EB and EC, have been used for inducing synchronized ovulation in beef cows. Administration of EC at the time of P4 device removal on Day 8 or EB on Day 9 resulted in ovulation at 68.5 and 70.2 hours after P4 device removal, respectively (Sales et al., 2012). In addition, the P/AI was similar between cows treated with EB on Day 9 (57.5%; 277/482) and EC on Day 8 (61.8%; 291/471; Sales et al., 2012). The traditional protocol in which EB is administered on Day 9, however, requires one extra handling (Day 0, EB + P4 device insertion; Day 8, PGF2α + P4 device removal; Day 9, EB; and Day 10, TAI), implying a disadvantage for reproductive management. To decrease the number of times, cows have to be handled, EB was administered at the time of P4 device removal. This administration of EB at the time of P4 device removal on Day 8 resulted in anticipated ovulation (59.4 hours after P4 device removal) and the resulting P/AI was satisfactory when TAI was performed 48 hours after P4 device removal (Ayres et al., 2008). The TAI 54 hours after P4 device removal resulted in a decreased P/AI, probably because TAI was performed near the time of the synchronized ovulation. Another report (Cavalieri et al., 2002) also described lower P/AI when EB was administered at the time of P4 device removal (Day 8) compared to EB administered 24 hours after device removal (Day 9). In another study, our group (Crepaldi et al., 2019), aimed to minimize the number of handlings during protocols for TAI in beef cows treated for induction of ovulation with EB at the time of P4 device removal. In this study, EB administration and P4 device removal were performed 10 hours later (Day 8.5; EB8.5 group) than at the conventional time (Day 8). Thus, cows of the EB8.5 group were submitted to TAI 38 to 42 hours or 44 to 48 hours after P4 device removal (D10). The ovarian response and P/AI of *Bos indicus* cows were similar among treatment group [EB8.5 = 60.1 (200/333), EB on D9 = 66.7 (232/348) and EC on D8 = 66.0 (233/353)]. These outcomes were observed due to the distinct response for induction of a preovulatory LH surge release when E2 esters are used in the treatment protocol. In a previous study, cows submitted to TAI that were treated with EC for induction of ovulation had a preovulatory release of LH surge 31 hours later than cows treated with EB (Sales et al., 2012). This difference in timing of the pre-ovulatory release of LH surge resulted in an expected delay of 10 hours at the time of ovulation. The delay by 10 hours in EB administration and P4 device removal, as compared with the timing when there are typically administrations of EB in the EB8.5 group, resulted in a day and timing of ovulation (Day 11 AM) similar to the EC administered on Day 8 (AM) and EB administered on Day 9 (AM). This adjustment in time of EB treatment and P4 device removal allowed the TAI to occur in the morning and afternoon of day 10 of the treatment protocol without hampering reproductive efficiency.

#### Time of artificial insemination (TAI)

Several groups have studied the appropriate timing of AI relative to the onset of estrus or ovulation in cows (Pursley et al., 1998; Dransfield et al., 1998; Roelofs et al., 2006). The general consensus is that later AI (>12 hours after the onset of estrus) usually results in greater fertilization rates but lower embryo quality when compared to insemination closer to the onset of estrus (Dalton et al., 2001; Saacke, 2008). For example, a large field study that included 17 herds and 2,661 breedings demonstrated that inseminating >24 hours after the onset of estrus resulted in a dramatic reduction in the frequency of pregnancy compared to inseminations performed between 4 and 12 hours after the onset of estrus (Dransfield et al., 1998). For TAI protocols, the time of insemination depends on the ovulation inducer used. Usually, TAI is performed 48 to 54 hours after EC administration on D8, 24 to 30 hours after EB administration on D9 (Sales et al., 2012), and 16 hours after GnRH administration (Wiltbank et al., 2015).

Unfortunately, the optimal interval for TAI with non-sorted sperm may not be compatible with the use of sex-sorted sperm for several reasons, including the potentially reduced lifespan of sex-sorted sperm in the female reproductive tract (Maxwell et al., 2004), fewer numbers of sorted sperm/straw (DeJarnette et al., 2008) and possible pre-capacitation induced by the sorting procedure (Lu and Seidel, 2004). In a small field trial, it has been reported an increased P/AI in heifers receiving AI 18–24 hours after the observed onset of estrus, as compared to those inseminated at 0–12 hours (Schenk et al., 2009). It is therefore reasonable to expect that decreasing the insemination-ovulation interval may be critical for achieving greater P/AI with sex-sorted sperm following TAI. A study conducted by our research group verified that increasing the interval between P4 device removal and TAI, such that most cattle were bred 0 to 12 hours before the synchronized ovulation, improved P/AI in TAI programs using sex-sorted sperm (Sales et al., 2011b).

Some strategies are used at the time of AI to optimize the response to the TAI protocol in *Bos indicus* cows. The use of GnRH at the time of TAI increased P/AI in cows that did not show estrus (52.7 [n = 393] vs. 38.1% [n = 420]; P = 0.001), in cows with BCS < 3.0 (57.1 [n = 723] vs. 48.6% [n = 698]; P = 0.001), and in primiparous cows (50.1 [n = 465] vs. 41.9% [n = 497]; P=0.001 Alves et al., 2021). Another alternative is the use of hCG, a glycoprotein hormone that has similar activity to LH. However, it has a longer half-life. In bovine females, its use can induce ovulation by binding to LH receptors in the granulosa and theca cells in ovarian follicles (De Rensis et al., 2008). In a recent study by our research group (Teixeira et al., 2022), the use of hCG increased P/AI in cows that did not show estrus (Control = 42.9% vs. hCG = 53.3%; P=0.04). Furthermore, the pregnancy at natural breeding tended to be greater in cows that showed estrus and received hCG (P=0.09).

## Previous exposure to injectable P4 in TAI protocol

In *Bos indicus* lactating beef cows, the post-partum anestrous period is long (Ruiz-Cortés and Olivera-Angel, 1999), negatively affecting the herd's productive and reproductive indexes (Montiel and Ahuja, 2005). Despite the benefits of the TAI protocols, part of the cows do not respond to the synchronization of ovulation protocols due to a drastic reduction in LH pulse-frequancy observed mainly in primiparous cows (Sales et al., 2016) and in undernourished cows with low BCS (Grimard et al., 1995; Diskin et al., 2003).

**Figure 1:**
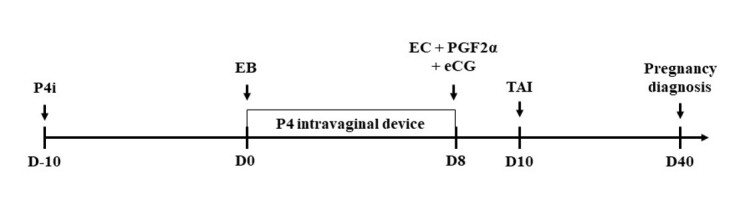
Progesterone-based timed-AI (TAI) protocol used in Brazil in suckled *Bos indicus* cows. P4i: 150 mg of injectable P4; Intravaginal device containing 1 g of progesterone; EB: 2 mg of estradiol benzoate; EC: 1 mg of estradiol cypionate; PGF2α: 500 µg of cloprostenol sodium; eCG: 300 IU of equine chorionic gonadotropin; TAI: Timed artificial insemination.

In postpartum *Bos indicus* cows, it is necessary to stimulate the hypothalamus to release GnRH and to increase LH pulse-frequency which would allow for the final growth of the dominant follicle and ovulation. The positive effects of ovulation synchronization protocols in anestrous cows are mainly due to the stimulation of exogenous P4 on the pulsatility of GnRH and LH (Rhodes et al., 2002), allowing for ovulation of a pre-ovulatory follicle early in postpartum (Baruselli et al., 2017). During the early postpartum period, circulating P4 reduces the expression of E2 receptors in the hypothalamus by interfering with the hormone receptor-negative feedback in GnRH secretion (Day, 2004). However, in underfed cows with low BCS or primiparous, the final growth of the dominant follicle is hampered, resulting in small follicles at the time of TAI and 21% of cows do not respond to TAI protocols (Sales et al., 2016). Thus, in females that do not respond to the TAI protocol, the period of exposure to P4 during the ovulation synchronization protocol may not be sufficient to increase the LH pulsatility needed for ovulation to occur. Thus, treatment with P4 in anestrous cows increased follicular fluid E2 concentration due to increased LH pulse-frequency and its LH receptors on granulosa and theca cells in pre-ovulatory follicles (Rhodes et al., 2002). Some studies have shown that the use of P4 stimulates return of cyclicity in lactating dairy cows (Lucy et al., 2001). Recently, our research group conducted studies to evaluate the effect of injectable P4 (P4i) on the reproductive efficiency of postpartum *Bos indicus* and *Bos taurus* cows submitted to TAI. In the first study (Simões et al., 2018) the effect of previous exposure to P4i in TAI protocols on follicular growth and P/AI of postpartum *Bos indicus* cows was evaluated. In this study, the cows received 150 mg of P4i 10 days before the TAI protocol (D-10; Figure 1). The P4i treatment increased the follicular diameter at the beginning of the TAI protocol and on the day of removal of the P4 device. In addition, cows receiving P4i were 1.68 times more likely to become pregnant after TAI than the control group. Similar outcomes were observed in *Bos taurus* beef cows (Simões et al., 2024), in which the P4i treatment previous to TAI protocol increased P/AI (Control 45.6% [118/259)] and P4i 54.8% [142/259]; P=0.03). In another study (Santos et al., 2018) using 988 postpartum Nelore cows in adequate BCS (~3.0), a P4i treatment preceding the ovulation synchronization protocol did not influence P/AI (Control 64.7% [322/498] and P4i 62.9% [308/490]; P = 0.55) and cyclicity 30 days after TAI (Control 39.8% [70/176] and P4i 39.6% [72/182] P = 0.78). Thus, probably in cows with adequate BCS, postpartum LH pulsatility should allow growth and ovulation of a preovulatory follicle. This difference in fertility after P4 treatment is probably due to the BCS of the animals in the different studies. In the study by Simões et al. (2018), the cows were nutritionally impaired which resulted in low BCS. Nutritionally deficient cows have lower postpartum LH pulsatility associated to the formation of metabolites (Nonesterified fatty acids, Beta-hydroxybutyrate and acetate), endorphins and peptides (mainly neuropeptide Y) known to produce negative feedback, blocking hypothalamic GnRH release (Hess et al., 2005). Thus, treatment with P4i before ovulation synchronization protocols may have increased LH secretion (Day, 2004), which resulted in greater P/AI. Similar outcomes were observed in *Bos taurus* dairy cows (Simões et al., 2023), in which the P4i treatment previous to TAI protocols increased P/AI (30 days: Control - 52.1% [122/234] and P4i – 56.2% [144/256]; and 60 days: Control - 49.6% [115/232] and P4i - 53.5% [136/254]). In addition, in the subgroup of cows without CL on D-7, the P/AI at 30 and 60 days after TAI was greater in cows of the P4i group (30 days: Control-NoCL - 32.7% [17/52] and P4i-NoCL - 50.7% [35/69]; P = 0.04; and 60 days: Control-NoCL 31.2% [15/48] and P4i-NoCL 50.0% [33/66]; P = 0.05). In this study, the cows received 300 mg of P4i 7 days before of the TAI protocol (D-7).

## Conclusion

In the last 40 years, TAI was the main ART used that has enabled the expansion of AI in dairy and beef farms in Brazil, increasing service rates and genetics. The TAI protocols in beef and dairy cows are well established and hormonal manipulation of follicular and luteal dynamics in ovulation synchronization programs for TAI consist of synchronizing the emergence of a new wave of follicular growth, controlling the length of the P4 phase by progestogens e prostaglandins and steroids (PGF2α and estrogens) and inducing the synchronized ovulation of the dominant follicle. Recently, several fine-tuning adjustments in the protocols, such as the P4i strategy (previous exposure to injectable P4) brought a significant increase in fertility in *Bos indicus* and *Bos taurus* females.
